# *Echinococcus granulosus sensu stricto*: silencing of thioredoxin peroxidase impairs the differentiation of protoscoleces into metacestodes

**DOI:** 10.1051/parasite/2018055

**Published:** 2018-11-26

**Authors:** Hui Wang, Jun Li, Chuanshan Zhang, Baoping Guo, Qin Wei, Liang Li, Ning Yang, Donald Peter McManus, Xiaoli Gao, Wenbao Zhang, Hao Wen

**Affiliations:** 1 Branch of The First Affiliated Hospital of Xinjiang Medical University Changji Xinjiang 831100 PR China; 2 State Key Laboratory of Pathogenesis, Prevention, Treatment of High Incidence Diseases in Central Asia, Clinical Medicine Institute, The First Affiliated Hospital of Xinjiang Medical University Urumqi Xinjiang 830054 PR China; 3 Molecular Parasitology Laboratory, Infectious Diseases Division, QIMR Berghofer Medical Research Institute Brisbane Queensland 4006 Australia; 4 Pharmacy College of Xinjiang Medical University Urumqi 830011 PR China

**Keywords:** *Echinococcus granulosus sensu stricto*, Protoscoleces, *EgTPx*, Antioxidant defence, RNA interference, siRNA, Development

## Abstract

Cystic echinococcosis (CE) is a cosmopolitan parasitic disease caused by infection with the larval stage of *Echinococcus granulosus sensu lato*. Thioredoxin peroxidase (TPx) may play an essential role in the antioxidant defence system of *E. granulosus s.l.* as neither catalase nor glutathione peroxidase activities have been detected in the parasite. However, it is not known whether TPx affects the survival and growth of *E. granulosus s.l.* during development. In this study, three fragments of siRNA specific for *EgTPx* (siRNA-1/2/3) were designed and transfected into protoscoleces of *E. granulosus sensu stricto* by electroporation. Quantitative real-time PCR and Western blotting analysis showed that siRNA-3 significantly reduced the expression of *EgTPx.* Coincidentally, knockdown of *EgTPx* expression in protoscoleces with siRNA-3 significantly reduced the viability of the parasite under oxidative stress induced by 0.6 mM H_2_O_2_. *In vitro* culture studies showed that protoscoleces treated with siRNA-3 reduced pre-microcyst formation. *In vivo* experiments showed that injecting mice intraperitoneally with protoscoleces treated with siRNA-3 resulted in a significant reduction in the number, size and weight of CE cysts compared with those of control animals. Silencing of *EgTPx* led to the impairment of growth of *E. granulosus s.s.* both *in vitro* and *in vivo*, indicating that *EgTPx* is an important factor for protoscoleces survival and plays an important role in the antioxidant defence against the host during development.

## Abstract (fr)

L’échinococcose cystique (EC) est une maladie parasitaire cosmopolite causée par une infection par le stade larvaire d’*Echinococcus granulosus sensu lato*. La thiorédoxine peroxydase (TPx) peut jouer un rôle essentiel dans le système de défense antioxydant d’*E. granulosus s.l.*, comme aucune activité de la catalase ni de la glutathion peroxydase n’a été détectée chez ce parasite. Cependant, on ignore si la TPx a des effets sur la survie et la croissance d’*E. granulosus s.l.* pendant le développement. Dans cette étude, trois fragments d’ARNsi spécifiques de EgTPx (siRNA-1/2/3) ont été conçus et transfectés dans des protoscolex d’*E. granulosus sensu stricto* par électroporation. La PCR quantitative en temps réel et l’analyse par Western blot ont montré que siRNA-3 réduisait significativement l’expression d’EgTPx. De même, l’inactivation de l’expression de EgTPx dans les protoscolex avec siRNA-3 a considérablement réduit la viabilité du parasite sous un stress oxydatif induit par 0,6 mM H_2_O_2_. Des études de culture *in vitro* ont montré que les protoscolex traités avec le siRNA-3 diminuaient la formation de pré-microcystes. Des expériences *in vivo* ont montré que des souris ayant reçu une injection de protoscolex traités par siRNA-3 par voie intrapéritonéale montraient une réduction significative du nombre, de la taille et du poids des kystes d’EC par rapport à ceux des animaux témoins. Le silençage d’EgTPx a entraîné une réduction de la croissance d’*E. granulosus sensu stricto* à la fois *in vitro* et *in vivo*, ce qui indique que EgTPx est un facteur important pour la survie des protoscolex et joue un rôle important dans la défense anti-oxydante contre l’hôte au cours du développement.

## Introduction

Cystic echinococcosis (CE), caused by the larval stage of *Echinococcus granulosus sensu lato* (*E. granulosus s.l.)*, is a cosmopolitan parasitic zoonosis that has a considerable impact on public health and animal production worldwide [3, 51]. Currently, there are more than 3 million echinococcosis patients [9, 24], and the disease causes over US$ 3 billion in economic losses each year [1]. The prevalence of the disease ranges from 0.8% to 11.9% in Tibetan communities in western China [51], and from 4.7% to 9.1% in Peru [12, 28]. CE is difficult to treat due to the lack of an effective drug [23, 50]. Consequently, there is an urgent need for a new chemotherapeutic agent for the treatment of CE.

Cystic echinococcosis is characterized by unilocular, fluid-filled cysts mainly located in the liver and lungs of patients [2]. Echinococcal cysts can survive in patients for over 50 years without apparently causing pathological damage in host tissues surrounding the cysts [44, 48], indicating that *E. granulosus s.l.* has evolved protective mechanisms which underpin this long-term survival. Naturally, *E. granulosus s.l.* cysts live in an aerobic environment in the mammalian host and the parasites are constantly at risk of attack by reactive oxygen species (ROS) produced by either the host or the cysts themselves [11]. To protect themselves from the damage caused by ROS, the parasites have developed efficient defense systems of enzymatic antioxidants for survival [22]. The reduction of ROS is highly dependent on superoxide dismutase (SOD), catalase, glutathione peroxidase (Gpx) and peroxiredoxin (Prx): SOD which converts superoxide ($O_{2}^{-}$) to hydrogen peroxide (H_2_O_2_), and then catalase, Gpx and Prx which detoxify H_2_O_2_ [39]. Given that no catalase or GPx activities are detectable in *E. granulosus s.l.*, Prx is the main H_2_O_2_-detoxifying enzyme in this parasite [36].

Peroxiredoxins (Prxs) are ubiquitous cysteine (Cys)-dependent peroxidases and are widely distributed among helminth and protozoan parasites [13]. Three Prx genes have been reported in parasitic platyhelminth genomes; two of these encode cytosolic proteins and the third encodes a mitochondrial protein [41]. These Prxs have been identified in *E. granulosus s.l.*, namely thioredoxin peroxidase (TPx), peroxiredoxin 2 and thioredoxin dependent peroxide reductase [45]. TPx, a typical 2-Cys Prx, was first cloned from a bovine strain of *E. granulosus s.l.* from Uruguay (referred to as *EgTPx*) [36]. Sequence analysis showed that *EgTPx* belongs to the Prx1 subfamily (Prx1a, one of the two cytosolic Prxs’s) which accounts for over 80% of the Prxs in parasites, and is the largest Prx class overall [13]. In previous work, we showed that *EgTPx* is especially highly expressed in *E. granulosus s.l.* protoscoleces (PSC) [17] and EgTPx proteins can be recognized in sera from 83% of confirmed CE patients [20]. Recent proteomic analysis [7, 10, 26, 27, 42, 46] and transcriptomics profiling [31, 45, 53] showed that EgTPx is an abundant excretory-secretory protein released by PSC, suggesting that it plays a potentially pivotal role at the host-parasite interface. However, the role of *EgTPx* in *E. granulosus s.l.* growth and development is not well defined.

RNA interference (RNAi) has been applied to a range of parasitic helminths to characterise gene function in worm growth [6], including *Schistosoma* species [5, 8, 14], *Fasciola hepatica* [21] and cestodes [25, 32, 33, 43]. However, the application of RNAi to *E. granulosus s.l.* has not been well explored. In the present study, we used RNAi-mediated gene silencing to investigate the role of *EgTPx* in the growth and development of *E. granulosus sensu stricto* (*E. granulosus s.s.*). We show that silencing *EgTPx* expression in PSCs results in a decrease in the ability of PSCs to counteract oxidative stress and impairs their ability to develop into metacestodes both *in vitro* and *in vivo.*

## Material and methods

### Ethics statement

Female BALB/c mice (6–8 weeks of age) were purchased from Beijing Vital River Laboratory Animal Technology Company Limited, and housed in specific pathogen-free (SPF) facilities at the First Affiliated Hospital of Xinjiang Medical University (FAH-XMU). Experimental protocols for using mice were approved by the Ethics Committee of the FAH-XMU (Approval No. 20170809-01).

### Preparation of parasite materials

PSCs were removed aseptically from sheep liver hydatid cysts collected from a slaughterhouse in Urumqi, Xinjiang, China. Briefly, PSCs were isolated under sterile conditions from intact cysts, and then digested with 1% (w/v) pepsin, pH 2.0 for 30 min at 37 °C to remove immature PSCs. After digestion, PSCs were washed five times with sterile phosphate-buffered saline (PBS) containing 100 U/mL penicillin/100 μg/mL streptomycin and maintained in RPMI1640 culture medium (Gibco, Auckland, New Zealand) at 37 °C [35, 47, 52]. PSC viability was determined by staining with 0.1% methylene blue. Only PSC samples with ≥95% viability were employed for further processing. The *E. granulosus s.s.* genotype was determined by analysing the mitochondrial cytochrome oxidase 1 (*cox1*) gene sequence amplified from the PSC genomic DNA.

### Preparation of mouse polyclonal antibody against the recombinant EgTPx protein

The cDNA sequence encoding *EgTPx* (GenBank No. AF478688) was amplified using the primer sets: 5′-GCGAATTCATGGCTGCTGTTGTT-3′ and 5′- ACAGCGGCCGCCTATCACGAGCTCATGAA-3′ with restriction enzyme sites for *Eco*RI and *Not*I, respectively. The PCR product was digested with *Eco*RI and *Not*I and then inserted into the pET-28a expression vector and transformed into competent *Escherichia coli* BL21 (DE3) cells. Expression of the recombinant EgTPx (rEgTPx) protein was induced with 0.4 mM isopropyl thio-*β*-D-galactoside (IPTG) for 4 h of incubation. The expressed rEgTPx protein was purified by affinity chromatography using Ni–NTA resin (Qiagen, Germany) according to the manufacturer’s instructions. The purity of the purified rEgTPx protein was evaluated by 12% (w/v) sodium dodecyl sulphate polyacrylamide gel electrophoresis (SDS-PAGE) and subsequent Coomassie Brilliant Blue staining. The concentration of the expressed protein was measured using the BCA assay (Thermo Scientific, Rockford, IL, USA). An anti-serum against rEgTPx was developed by three-time immunisation (the first in Freunds Complete Adjuvant and the two remaining in Freunds Incomplete Adjuvant) of BALB/c mice with two-week intervals, and then the antiserum was adsorbed against a lysate of bacterial cells transformed with the pET-28a expression vector. The specificity of the mouse serum anti-rEgTPx was evaluated by Western blot and Dot-blot analysis.

### Determination of PSC viability under oxidative stress

PSCs were pre-cultured in RPMI 1640 medium for 4 h at 37 °C; then, immediately, H_2_O_2_ was added at different concentrations (0, 0.2, 0.4, 0.6, 0.8, 1.0, 2.0 mM) and the PSCs were cultured for 1 h, 2 h and 3 h. The PSCs were stained with 0.1% (v/v) methylene blue and the numbers of live parasites counted immediately under an inverted microscope (Olympus, Tokyo, Japan) to determine their viability.

### Preparation of short interfering RNA

Three fragments of siRNA targeting *EgTPx* were designed and synthesised by Guangzhou RiboBio Co., Ltd. (RiboBio, www.sirna.cn), each comprising 19 nt with an overhang (dTdT). The three siRNA compositions were: siRNA-1 (Sense: 5′-GGAACAACGUGAGCCGAAAdTdT-3′, Antisense: 5′-UUUCGGCUCCGUUGU UCCdTdT-3′); siRNA-2 (Sense: 5′-CCGUGCUGAUGAGUUUCAUdTdT-3′, Antisense: 5′-AUGAAACUCAUCAGCACGGdTdT-3′); siRNA-3 (Sense: 5′-GGUGGUGUUCAAGGUAUGAdTdT-3′, Antisense: 5′-UCAUACCUUG AACACCACCdTdT-3′). The sequence alignment of the three *EgTPx* siRNAs sequences is shown in Figure S1. A control fluorescent-labelled siRNA (siR-Ribo^TM^ Transfection Control-Cy3, synthesised by Guangzhou RiboBio Co., Ltd.) was used to determine the transfection efficacy of the PSCs. An siRNA (siR-Ribo^TM^ siRNA Negative Control, NC), which does not specifically target any human, mouse, rat or *E. granulosus s.l.* gene, was used as a negative control.

### siRNA delivery to PSCs of *E. granulosus s.s.*

Soaking and electroporation methods were used for transfecting siRNA into PSCs. In brief, for soaking, about 2000 PSCs were incubated for 2 h in 100 μL RPMI1640 medium containing Cy3-labelled control siRNA (5 μM final concentration). For electroporation, 2000 PSCs were washed three times with RNAi electroporation buffer (150 mM sucrose, 27 mM Na_2_HPO_4_, adjusted pH to 7.5) and then resuspended in 100 μL electroporation buffer containing Cy3-labelled control siRNA at a final concentration of 5 μM in a 4-mm electroporation cuvette. Electroporation was performed at 125 V, 20 ms, 1 pulse using a Square Wave Protocol (Gene Pulser II, Bio-Rad, USA). After incubation at 37 °C for 10 min, 1 mL culture medium was added and the PSCs were transferred to 24-well plates for further incubation at 37 °C in 5% CO_2_ in the dark. After 2 h treatment, the parasites were washed with PBS and examined under a Confocal microscope (TCS SP8, Leica).

### Viability of PSCs treated with target-siRNA *in vitro*

Five groups of PSCs were prepared, including three *EgTPx* siRNA-treated groups (siRNA-1/2/3), a negative control siRNA-treated group (NC), and an untreated group (Untreated). The PSCs were transfected with siRNA-1/2/3 or negative control siRNA using electroporation at a final concentration of 5 μM in 100 μL of electroporation buffer containing in each sample approximately 2000 PSCs in a 4-mm cuvette. The treated PSCs were then incubated for 3 days and collected to determine the effects of RNAi on *EgTPx* mRNA and protein levels. Meanwhile, the RNAi effects on the viability of the treated PSCs were evaluated using a suitable H_2_O_2_ concentration which PSCs could tolerate under normal culture conditions. Viability was calculated by counting the number of PSCs that were stained with 0.1% methylene blue. Finally, PSCs treated with the selected siRNA were incubated for a further two weeks in RPMI 1640 (Gibico) supplemented with 10% (v/v) heat-inactivated FBS (Gibico), 100 U/mL penicillin, and 100 μg/mL streptomycin (Hyclone) at 37 °C in an atmosphere of 5% CO_2_ in air to observe the formation of microcysts. Approximately half of the medium was changed every 3 days. Each treatment was carried out in triplicate and experiments were repeated twice using samples of PSCs collected at different times.

### Infectivity assay of PSCs treated with target-siRNA *in vivo*

To determine whether PSCs treated with the selected siRNA retained the ability to grow in *vivo*, three groups (*n* = 24/group) of BALB/c mice were used; these included a selected siRNA treated group (siRNA-3 Group), a negative control siRNA treated group (NC Group), and an untreated group (Untreated group). In brief, the selected siRNA and negative control siRNA were first transfected into PSCs by electroporation. Then, 2000 PSCs were injected intraperitoneally into each mouse in the three groups. After 1, 3, 6 and 9 months post-inoculation, 7–8 mice from each group were sacrificed for necropsy and parasite cysts were collected from the intraperitoneal cavity and the number counted. Serum alanine aminotransferase (ALT) and aspartate aminotransferase (AST) activity in the sera of treated and untreated mice were determined using ALT and AST Reagent Kits (Abcam, Cambridge, UK).

### Quantitative real-time PCR (qRT-PCR)

Total RNA was extracted from PSCs using TRIzol reagent (Invitrogen, CA, USA), as previously described [18]. The quality and concentration of RNA was confirmed and 1 μg of the RNA sample was treated with DNase I (Thermo, Waltham, MA, USA) for 30 min at 37 °C to remove genomic DNA contamination. cDNA was then produced from the treated RNA using a reverse transcription kit (Thermo), according to the manufacturer’s instructions. qRT-PCR was performed for gene expression using 2 μL of 1:5 diluted cDNA and was run in a thermocycler (iQ5 Bio-Rad, Hercules, CA, USA) with SYBR Green PCR premix (Qiagen, Hilden, Germany). The sense and antisense primers for *EgTPx* (AF478688) were 5′-CGACGGTGAACTCAAGGATGT-3′, and 5′-GCACGGTCGTTAAAAGCGATTATC-3′. Primers for *actin I* (L07773) as an internal control [41] were 5′-GTTGTGCTATGTGGCACTCGACT-3′ and 5′-CAATCCAGACAGAGTATTTGCGTTC-3′. All samples were run in triplicate using the following cycle parameters: 95 °C for 5 min; 40 cycles at 95 °C for 10 s and at 60 °C for 30 s. A melting point curve was analysed after the PCR by increasing the temperature from 65 to 95 °C (0.5 °C increments) to validate the PCR amplification specificity. Furthermore, the PCR amplification efficiency of the target gene *EgTPx* (*E* = 112.9%, *R* ^2^ = 0.994) and reference gene *actin I* (*E* = 108.1%, *R* ^2^ = 0.990) was established by standard curves. Cycle threshold (Ct) values were normalised to the reference gene *actin I* and analysed using the 2-^ΔΔ^Ct method. Each relative value was normalised to the untreated PSC sample.

### Western blot analysis

For protein level analysis, proteins were isolated from PSC samples as described [17] and protein concentration was measured using the BCA protein assay. The proteins were separated through 12% SDS-PAGE and then transferred onto polyvinylidene fluoride (PVDF) membranes (Millipore Corp., MA, USA). After blocking, the membranes were incubated with mouse anti-rEgTPx serum (1:1000) or rabbit anti-β-actin serum (Santa Cruz Biotechnology, CA, USA. 1:2000) overnight at 4 °C followed by incubation with AP-conjugated second antibody (Cell Signaling Technology, Danvers, MA, USA). Protein bands were visualised with a BCIP/NBT kit (Invitrogen, Carlsbad, CA, USA). The expression levels of the respective proteins were quantified using Quantity One software (Bio-Rad) after scanning of the membranes.

### Statistics

Statistical analysis was performed using GraphPad Prism 5.0 software (San Diego, CA, USA). Data were expressed as the mean ± standard error of the mean and analysed statistically using one-way ANOVA with Tukey’s multiple comparison test at each time point; *p* < 0.05 was regarded as statistically significant.

## Results

### Tolerance of PSCs against H_2_O_2_ and EgTPx expression under H_2_O_2_ suppression

**Figure 1.**
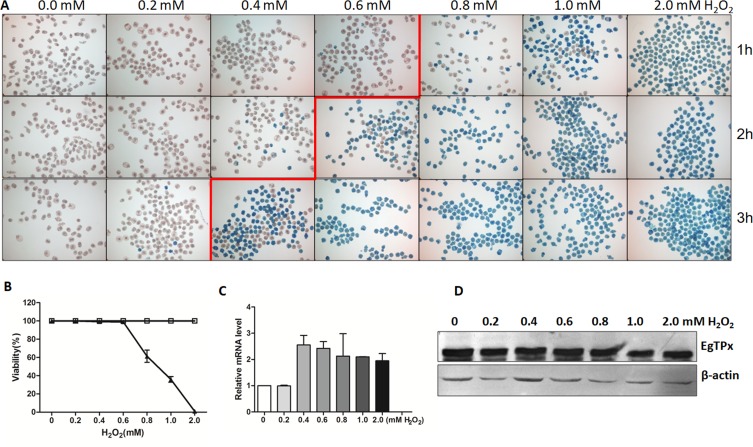
Viability and *EgTPx* expression of protoscoleces in the presence of H_2_O_2_. (A) Methylene blue staining of protoscoleces exposed to different H_2_O_2_ concentrations for 1 h, 2 h and 3 h. (B) Survival of protoscoleces incubated in medium with different concentration of H_2_O_2_ for 1 h (triangles). Protoscoleces incubated in medium without H_2_O_2_ were used as a control (open boxes), *n* ≈ 200. (C) Quantitative analysis of mRNA transcripts for expression of *EgTPx.* (D) Western blotting showing expression of EgTPx in different concentrations of H_2_O_2._ The bars indicate mean ± S.E.M.

To assess the tolerance of PSCs against H_2_O_2_, PSCs of *E. granulosus s.s.* (Genotype G1, common sheep strain) (Fig. S2) were collected and cultured in medium containing various concentrations of H_2_O_2_ for 1 h, 2 h and 3 h. The tolerance of PSC against H_2_O_2_ depended on the concentration of H_2_O_2_ and the time of processing. Methylene blue staining showed that *E. granulosus s.s.* PSCs were tolerant in medium containing 0.2 mM of H_2_O_2_; higher concentrations of H_2_O_2_ generally killed the parasites although they were able to survive for 1 h in medium containing 0.6 mM H_2_O_2_ (Figs. 1A and 1B). When cultured PSCs were incubated with H_2_O_2_ at different concentrations ranging from 0 to 2.0 mM, the mRNA level of *EgTPx* did not change significantly (Fig. 1C), and EgTPx protein levels were also not significantly increased (Fig. 1D).

### Transfection of siRNA into PSCs

**Figure 2.**
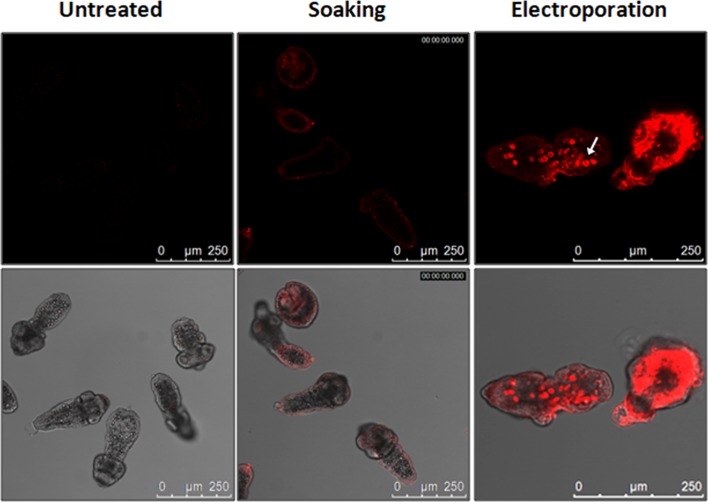
Transfection of *siRNA* into protoscoleces. Localisation of Cy3-labelled siRNA in protoscoleces following soaking or electroporation at 2 h was observed by confocal microscopy. The white arrow indicates a calcareous corpuscle. The scale bar represents 250 μm.

To optimise the transfection of siRNA into PSCs, two methods, soaking and electroporation, were used to deliver a Cy3-labelled siRNA into parasites. As expected, fluorescence was not detectable in the untreated PSCs. The soaking method resulted in a low level of transfection as only weak fluorescence was detected in the tegument of PSCs. In contrast, electroporation resulted in high efficacy for transfecting Cy3-labelled siRNA into PSCs with the average transfection efficiency being 79%. Notably, a high density of fluorescence was detected in many, but not all, calcareous corpuscles in PSCs (Fig. 2, Additional File 1). The experiment was repeated twice using PSC samples collected at different times.

### Down-regulation of *EgTPx* expression in PSCs treated with siRNAs

**Figure 3.**
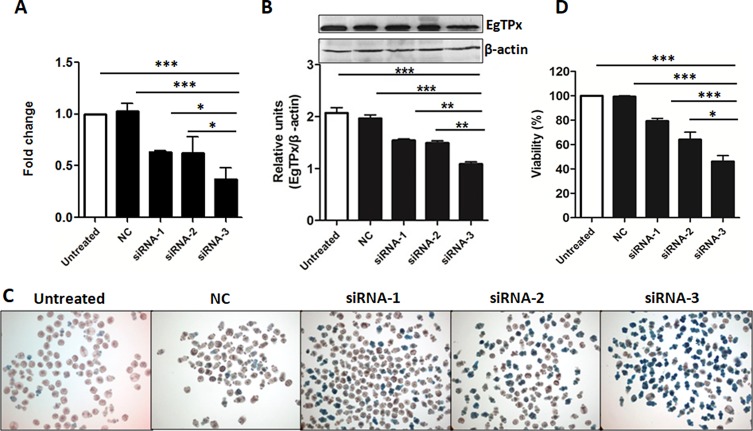
RNAi effects on *EgTPx* expression and viability of cultured protoscoleces. (A) Quantitative PCR analysis of *EgTPx* mRNA in siRNA-1/2/3-treated or untreated protoscoleces. (B) Western blotting analysis and quantification graph of EgTPx protein expression in siRNA-1/2/3-treated or untreated protoscoleces. (C), (D) RNAi effects on the viability of siRNA-treated protoscoleces under oxidative stress (H_2_O_2_, 0.6 mM, 1 h) by day 3. Bars represent the standard deviation. *p < 0.05; **p < 0.01; ***p < 0.001.

Both qRT-PCR and Western blot analysis were used to determine whether the targeted siRNAs affected the mRNA and protein expression levels of *EgTPx* in PSCs. qRT-PCR showed that the *EgTPx* transcription level in PSC treated with 5 μM of siRNA-1/2/3 was reduced significantly at day 3 post-electroporation (Untreated *vs*. NC *vs*. siRNA-1/2/3 PSC: 1.14 ± 0.24 *vs.* 1.03 ± 0.10 *vs*. 0.63 ± 0.01, 0.63 ± 0.22, 0.37 ± 0.15, *F*(4, 14) = 28.25, *p* < 0.0001) (Fig. 3A). In order to analyse the effects of the targeted siRNAs on the protein expression of EgTPx in PSC, mouse antiserum against purified rEgTPx was produced and purified. SDS-PAGE analysis showed a single purified rEgTPx band, with a molecular weight of approximately 25 kDa. Dot-blot analysis showed that the purified antiserum was specific to rEgTPx, as lysates of pET-28a expression bacterial cells were not recognised (Fig. S3). Western blot analysis showed that the native EgTPx protein band (~22 kDa) was the most pronounced band expressed in PSCs, although other bands with molecular sizes less than or larger than 22 kDa in PSCs were also detected, which may indicate either partial degradation of the native protein and/or be due to the fact that EgTPx itself contains two Cys which can form dimers with other worm proteins. This analysis showed that the protein expression of EgTPx was down-regulated in PSCs treated with 5 μM siRNA-3 at day 3 post-electroporation (Fig. 3B). Among the siRNAs, siRNA-3 was the most effective in terms of reducing *EgTPx* expression in PSCs.

### Silencing of *EgTPx* expression inhibits the viability of PSCs *in vitro*

To determine whether *EgTPx* gene knockdown by siRNA had an impact on the survivability of the treated PSCs under oxidative stress, three synthesised siRNAs (siRNA-1/2/3) and negative control siRNA were transfected into PSCs by electroporation. After 3 days of transfection, all samples were incubated with 0.6 mM H_2_O_2_ for 1 h; the viability of PSCs transfected with 5 μM of siRNA-1/2/3 was reduced to 79.29 ± 4.79%, 64.03 ± 13.69%, and 46.13 ± 10.84% (*F*(4, 21) = 29.73, *p* < 0.0001), respectively, compared with untreated PSCs (Figs. 3C and 3D). No statistical difference in PSC viability was observed between the untreated and negative control PSCs.

**Figure 4.**
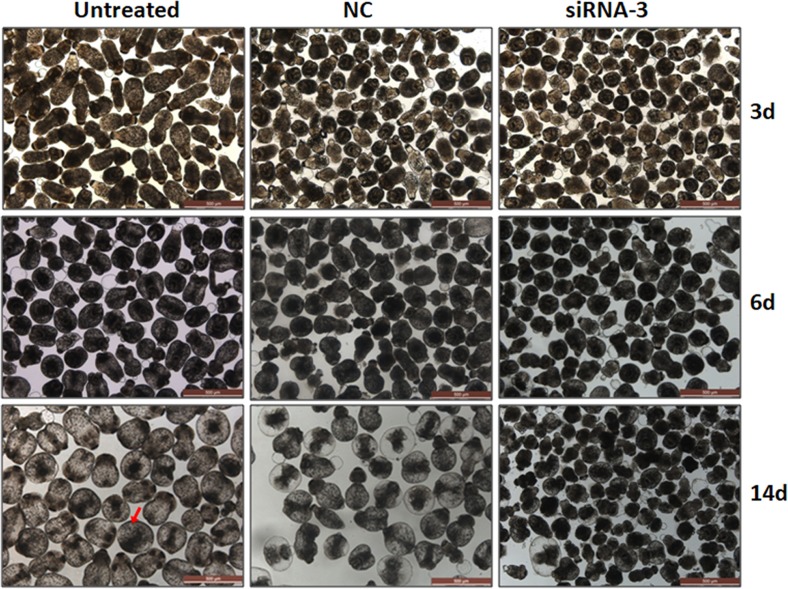
Silencing of *EgTPx* expression reduces the ability of protoscoleces cultured in vitro to develop into microcysts. Protoscoleces were incubated for 14 days to observe cyst formation after treatment with siRNA. The red arrow indicates a microcyst. The scale bar represents 500 μm.

To determine whether *EgTPx* gene knockdown by the most effective siRNA (siRNA-3) affected the development of PSCs under normal culture conditions, PSCs were first treated with 5 μM of siRNA-3 and then incubated for another two weeks; this resulted in a 92% reduction in PSCs developing to pre-microcysts compared with untreated PSCs (Fig. 4), indicating that siRNA-3 silencing arrested the differentiation and development of PSCs in a cystic direction.

### Silencing of *EgTPx* expression attenuates the infectivity of PSCs in mice

**Figure 5.**
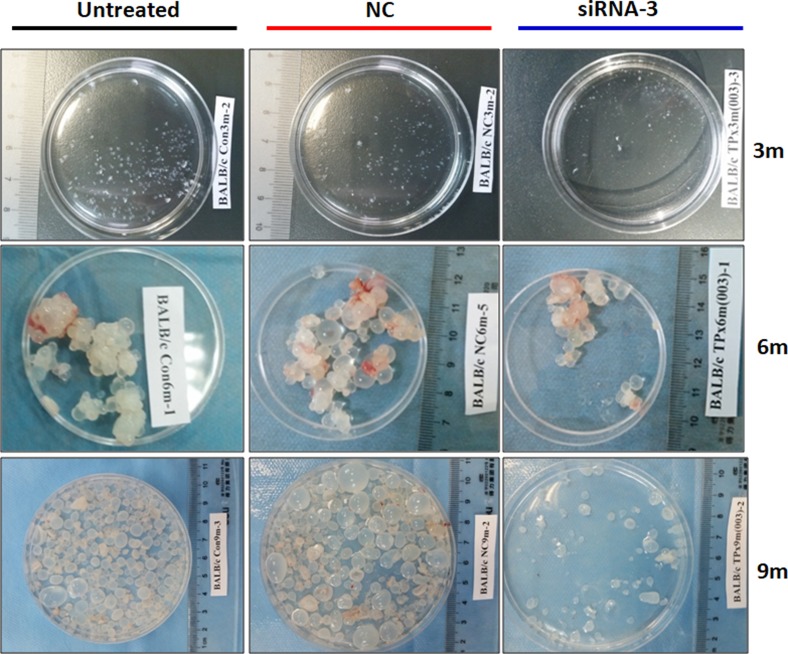
General observations of hydatid cysts collected from different treated groups at 3 months, 6 months and 9 months post-challenge infection. *n* = 7–8 mice/group, in two separate experiments.

**Figure 6.**
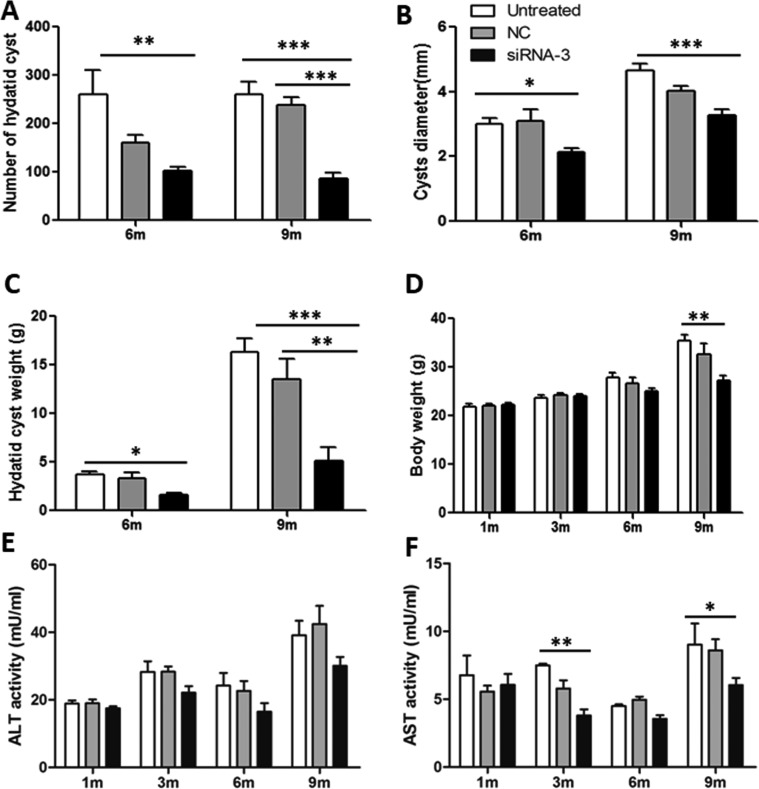
Silencing of *EgTPx* expression attenuates the infectivity of protoscoleces in mice. (A)–(C) Effects of siRNA-3 treatment on the mean number, size and weight of hydatid cysts collected from mice, respectively; (D) Effects of siRNA-3 treatment on the body weight of mice. (E), (F) Effects of siRNA-3 treatment on serum ALT and AST levels at 1, 3, 6 and 9 months post protoscoleces challenge infection. *p < 0.05; **p < 0.01. *n* = 7–8 mice/group, in two separate experiments.

To determine whether PSCs treated with siRNA-3 retained their ability to grow *in vivo*, siRNA-3 was first transfected into 2000 PSCs and the treated PSCs were then injected into mice intraperitoneally. Six months post-infection, the number of hydatid cysts present was significantly decreased compared with the cyst number from control mice (Untreated Group *vs*. NC Group *vs*. siRNA-3 Group: 261 ± 131 *vs*. 161 ± 41 *vs*. 102 ± 23 (*F*(2, 20) = 7.01, *p* = 0.0056). In addition, the cyst size (Untreated Group *vs*. NC Group *vs*. siRNA-3 Group: 3.02 ± 0.4 *vs*. 3.1 ± 0.89 *vs*. 2.12 ± 0.29 mm (*F*(2, 20) = 4.037, *p* = 0.0433) and total cyst weight (3.71 ± 0.73 *vs*. 3.31 ± 1.31 *vs*. 1.63 ± 0.46 g (*F*(2, 20) = 3.915, *p* = 0.0413) were also reduced. Nine months post-infection, the number of cysts (Untreated Group *vs*. NC Group *vs*. siRNA-3 Group: 261 ± 67 *vs*. 239 ± 41 *vs*. 87 ± 29 (*F*(2, 20) = 26.89, *p* < 0.0001), cyst size (Untreated Group *vs*. NC Group *vs*. siRNA-3 Group: 4.67 ± 0.45 *vs*. 4.03 ± 0.42 *vs*. 3.29 ± 0.46 mm (*F*(2, 20) = 15.912, *p* < 0.0001) and cyst weight (Untreated Group *vs*. NC Group *vs*. siRNA-3 Group: 16.36 ± 3.63 *vs*. 13.5 ± 5.57 *vs*. 5.09 ± 3.76 g (*F*(2, 20) = 12.35, *p* = 0.0004) were also significantly decreased. The body weight of mice infected with PSCs silenced with siRNA-3 (27.15 ± 2.84 g) was significantly lower than the body weight of mice treated with NC siRNA (32.61 ± 5.70 g) and untreated animals (35.38 ± 3.21 g) 9 months post-infection (*F*(2, 20) = 7.228, *p* = 0.005) (Figs. 5, 6A–6D).

The level of AST activity was also significantly lower in siRNA-3-treated mice (5.97 ± 1.19 mU/mL) compared with NC siRNA-treated (8.61 ± 1.62 mU/mL) and untreated animals (9.03 ± 3.09 mU/mL) 9 months post-infection (*F*(2, 20) = 4.415, *p* = 0.0422) (Figs. 6E and 6F).

## Discussion

Detoxification of peroxides in parasitic Platyhelminthes relies on three Prxs and a GPx, which are able to reduce H_2_O_2_ to H_2_O and O_2_ [49]. As neither GPx nor catalase are active in *E. granulosus s.l.* H_2_O_2_ in this parasite is mainly reduced by EgTPx [36] (a cytosolic Prx), which in turn is reduced by thioredoxins (Trx) and then the oxidised form of Trx is finally reduced by thioredoxin glutathione reductase (TGR) [45]. These components form an important antioxidant defence system in *E. granulosus s.l.* In the present study, we used siRNA to knockdown the transcription of *EgTPx*, an important H_2_O_2_ detoxifying enzyme in the *E. granulosus s.l.* antioxidant system, resulting in increased sensitivity towards H_2_O_2_ and severely impaired growth of the PSCs in a cystic direction both *in vitro* and *in vivo*. This indicates *EgTPx* is an important factor for PSC survival, playing an important role in oxygen defence during development.

RNA interference (RNAi) has been used successfully as a means of silencing specific gene expression to elucidate function and to assess the therapeutic value of candidate genes in helminths, including turbellarians [38], trematodes [19] and a monogenean [30], but there is relatively limited information on the use of the approach to determine gene function in cestodes. In terms of transfection methods, soaking and electroporation, or a combination of the two approaches, have been applied in *Moniezia expansa* [32] and *E. multilocularis* [25], with electroporation being the favoured method. In this study, we used both soaking and electroporation to transfect siRNA into PSCs of *E. granulosus s.s.* and showed that soaking was also useful for transfecting small RNA into PSCs. However, we found that fluorescence was only detected in the tegument of PSC treated by soaking. In contrast, in parasites subjected to electroporation, strong fluorescence was detected in many calcareous corpuscles in PSCs after treatment, possibly because the electroporation shock may have temporarily opened channels on the tegumental surface of PSCs or destabilised membranes, resulting in the formation of membrane pores facilitating increased entry of siRNA or dsRNA into the worm tissues.

It is noteworthy that *EgTPx* is also specifically expressed in many calcareous corpuscles in PSCs [17]. Calcareous corpuscles may act as a repository for EgTPx, whereby the enzyme can be released rapidly and in sufficient quantity to assist *E. granulosus s.l.* in escaping oxidative damage. Consequently, we considered that introducing *EgTPx*-specific siRNA into PSCs by electroporation may more effectively silence the expression of *EgTPx* and this was the case as *EgTPx* expression was down-regulated in PSCs treated with siRNAs by this method.

In addition, another notable outcome was that PSC with *EgTPx* silenced with the specific siRNAs had a lower survival rate than controls under oxidative stress induced by 0.6 mM H_2_O_2_, a concentration PSCs were able to tolerate under normal culture conditions; of these, siRNA-3 was shown to be the most efficient in reducing survival. This may be due to the fact that siRNA-3 may have a looser target site structure than the two other siRNAs, and its sequence composition may also facilitate increased binding to the *EgTPx* gene.

Reduction of Prx expression by RNAi has been demonstrated to affect the viability and growth of other parasitic worms, especially schistosomes, both *in vivo* and *in vitro* [15, 29, 40]. In addition, some recent studies have shown that inhibition of expression of TGR, another essential enzyme in the H_2_O_2_ detoxification system in platyhelminth parasites, by siRNA and specific TGR inhibitors also severely hampered *in vitro* development of *E*. *granulosus s.l.* [4, 34, 37] larvae into cysts and even killed larval worms of *S*. *mansoni* [16]. In our study, infection of mice with PSCs treated with siRNA-3 specific for *EgTPx* resulted in a significant reduction in worm burden compared to control infected mice. These results demonstrate the importance of *EgTPx* as well as *Eg*TGR for the survival of *E. granulosus s.l.* in the intermediate host.

In conclusion, we demonstrated that *EgTPx* is an important antioxidant gene that is essential for PSC survival and plays an important role in PSC development. The availability of an appropriate gene silencing tool will be useful for future studies of gene function in *E. granulosus s.s*. We showed that silencing of *EgTPx* significantly reduced the number of cysts developing from PSCs and also impaired the growth of cysts both *in vitro* and *in vivo*, which indicates that *EgTPx*-siRNA may have value as a protoscolecidal agent for the treatment of cystic echinococcosis. Our findings suggest that research efforts targeting the inhibition of members, such as TPx, TGR or Trx, of the antioxidant system in *E. granulosus s.l.* represent a promising strategy in the development of new anti-echinococcosis drugs.

## Supplementary Materials

**Figure S1.**
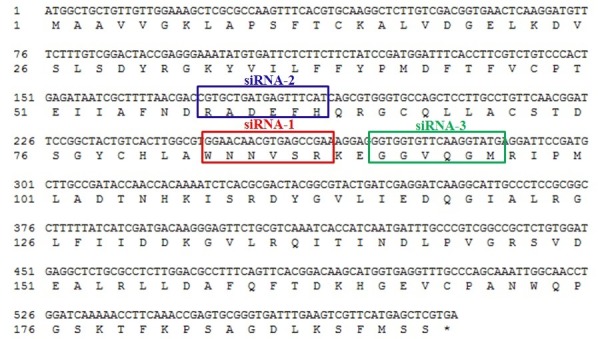
Sequence alignment of the three siRNAs with the *EgTPx* sequence.**Figure S2.**
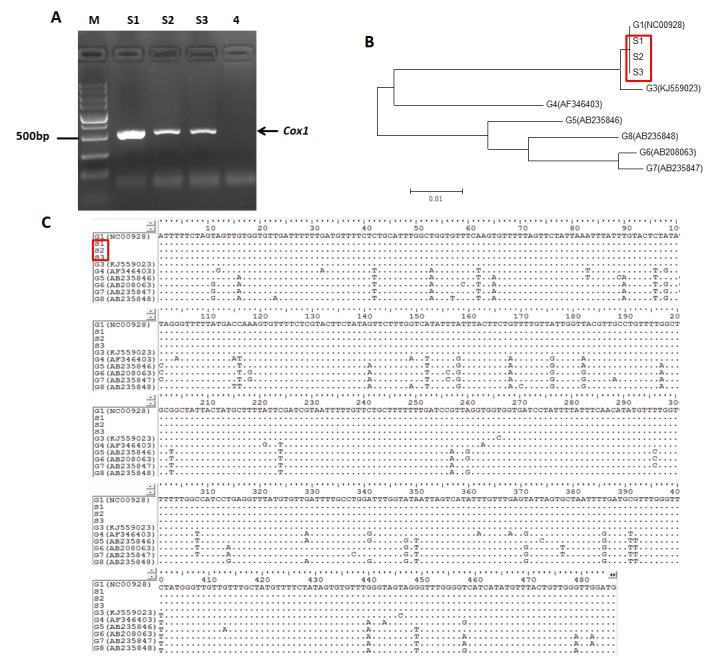
Genotypic characterisation of *Echinococcus granulosus s.l.* isolates using the cytochrome oxidase 1 (*cox1*) gene. *A*, Gel electrophoresis of amplified PCR products of protoscoleces used in this study. M: Indicates 5000 bp DNA Marker; Lane S1-3: protoscoleces samples; Lane 4: Negative control; *B*, Phylogenetic tree with the protoscoleces used in this study (denoted as S1–S3) based on the *cox1* gene, with reference sequences for 7 different genotypes from GenBank (G1 – NC00928, G3 – KJ559023, G4 – AF346403, G5 – AB235846, G6 – AB208063, G7 – AB235847a and G8 – AB235848); *C*, Alignment positions of the amplified mitochondrial *cox1* gene sequences in comparison with available published sequences of *Echinococcus granulosus s.l.* genotypes G1–G8. Dots indicate identity with the G1 (*Echinococcus granulosus s.s.*, common sheep strain) sequence chosen as the reference.**Figure S3.**
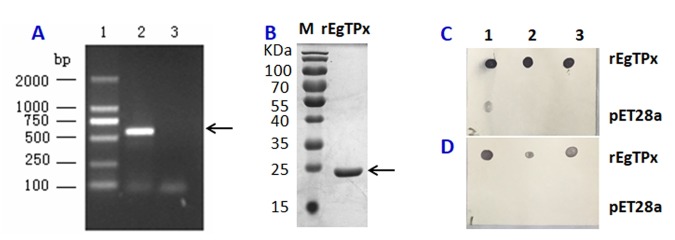
rEgTPx expression and Dot-blot analysis of the purified mouse anti-rEgTPx serum. *A*, Agarose gel electrophoresis image of the PCR-amplified EgTPx gene. Lane1: DNA Marker/DL2 000 (Takara, Otsu, Japan); Lane2: EgTPx was successfully amplified; Lane3: Without template as native control. *B*, SDS-PAGE analysis of rEgTPx. M: Protein markers; Lane 1: Purified rEgTPx protein. *C*, Dot-blot analysis of the EgTPx antiserum before purification. *D*, Dot-blot analysis of the EgTPx antiserum after purification; lanes 1, 2 and 3 show the one-third serial dilutions of purified rEgTPx (upper) and the lysates of bacterial cells transformed with the pET-28a expression vector expressing the His tag (lower).***Additional file 1: Video file 1.*** Fluorescence of Cy3-labelled siRNA in protoscoleces.Supplementary materials are available at https://www.parasite-journal.org/10.1051/parasite/2018055/olm

## Conflict of interest

The authors declare that they have no conflicts of interest in relation to this article.
